# Combination of Albumin-Globulin Score and Sarcopenia to Predict Prognosis in Patients With Renal Cell Carcinoma Undergoing Laparoscopic Nephrectomy

**DOI:** 10.3389/fnut.2021.731466

**Published:** 2021-09-23

**Authors:** Weipu Mao, Nieke Zhang, Keyi Wang, Qiang Hu, Si Sun, Zhipeng Xu, Junjie Yu, Can Wang, Saisai Chen, Bin Xu, Jianping Wu, Hua Zhang, Ming Chen

**Affiliations:** ^1^Department of Urology, Affiliated Zhongda Hospital of Southeast University, Nanjing, China; ^2^Department of Urology, Shidong Hospital of Yangpu District, University of Shanghai for Science and Technology, Shanghai, China; ^3^Department of Medical College, Southeast University, Nanjing, China; ^4^Department of Urology, School of Medicine, Shanghai Tenth People's Hospital, Tongji University, Shanghai, China; ^5^Department of Health Insurance, School of Public Health, Southeast University, Nanjing, China

**Keywords:** renal cell carcinoma, combination of albumin-globulin score, sarcopenia, albumin-globulin score, sarcopenia, prognostic indicator, nephrectomy

## Abstract

We conducted a multicenter clinical study to construct a novel index based on a combination of albumin-globulin score and sarcopenia (CAS) that can comprehensively reflect patients' nutritional and inflammatory status and assess the prognostic value of CAS in renal cell carcinoma (RCC) patients. Between 2014 and 2019, 443 patients from 3 centers who underwent nephrectomy were collected (343 in the training set and 100 in the test set). Kaplan-Meier curves were employed to analyze the impact of albumin-globulin ratio (AGR), albumin-globulin score (AGS), sarcopenia, and CAS on overall survival (OS) and cancer-specific survival (CSS) in RCC patients. Receiver operating characteristic (ROC) curves were used to assess the predictive ability of AGR, AGS, sarcopenia, and CAS on prognosis. High AGR, low AGS, and nonsarcopenia were associated with higher OS and CSS. According to CAS, the training set included 60 (17.5%) patients in grade 1, 176 (51.3%) patients in grade 2, and 107 (31.2%) patients in grade 3. Lower CAS was linked to longer OS and CSS. Multivariate Cox regression analysis revealed that CAS was an independent risk factor for OS (grade 1 vs. grade 3: aHR = 0.08; 95% CI: 0.01–0.58, *p* = 0.012; grade 2 vs. grade 3: aHR = 0.47; 95% CI: 0.25–0.88, *p* = 0.018) and CSS (grade 1 vs. grade 3: aHR = 0.12; 95% CI: 0.02–0.94, *p* = 0.043; grade 2 vs. grade 3: aHR = 0.31; 95% CI: 0.13–0.71, *p* = 0.006) in RCC patients undergoing nephrectomy. Additionally, CAS had higher accuracy in predicting OS (AUC = 0.687) and CSS (AUC = 0.710) than AGR, AGS, and sarcopenia. In addition, similar results were obtained in the test set. The novel index CAS developed in this study, which reflects patients' nutritional and inflammatory status, can better predict the prognosis of RCC patients.

## Introduction

Renal cell carcinoma (RCC), alternatively referred to as renal cancer, is 1 of the most prevalent malignancies of the urinary system. It is common cancer with morbidity of 2–3% in systemic malignant tumors and 80–85% in renal cancers (1). Due to its increasing incidence, 170,000 RCC patients died worldwide in 2018, with a mortality rate of ~ 2.7% (2). When RCC is early detected, it can be effectively treated with radical or partial nephrectomy, with a 5-year survival rate of 93% (3). However, over 30% of patients progress to advanced RCC at the first diagnosis, and 10–20% of patients with early RCC experience recurrence after treatments (4). Advanced RCC patients have a decreased 5-year survival rate of 67% due to regional and distant metastases (5).

Apart from the time of diagnosis, numerous other factors affect the prognosis of RCC patients, such as tumor size, pathological stage, and other biochemical indicators (6). Albumin (ALB) and globulin (GLB) are indicators of systemic nutritional status, and their ratio (AGR) is an independent prognostic factor for RCC patients (7). Albumin-globulin score (AGS) is another model based on ALB and GLB (8). However, no previous studies have investigated the relationship between AGR and AGS and long-term outcomes in RCC patients undergoing nephrectomy.

Sarcopenia is an emerging index of nutritious status, an extensive and progressive skeletal muscle disease characterized by loss of muscle mass and strength (9). Sarcopenia was assessed by measuring lumbar skeletal muscle index (SMI) and total psoas index (TPI) preoperatively using computed tomography (CT). Recently, sarcopenia was reported to be connected to inflammatory diseases, malignancies, and malnutrition (10). Sarcopenia, in particular, is a poor prognostic indicator in various tumors, including hepatocellular carcinoma, gastroesophageal tumor, colorectal cancer, and urothelial carcinomas (11), and our previous study found that sarcopenia is a risk factor for the survival time of cancer patients, including RCC and bladder cancer (12, 13).

This study aimed to determine the influence of AGR, AGS, and sarcopenia on the prognosis of RCC patients treated with laparoscopic nephrectomy and to build a novel index based on a combination of AGS and sarcopenia (CAS) that can more comprehensively reflect the nutritional and inflammatory status of RCC patients and investigate the prognostic ability of CAS in RCC patients undergoing laparoscopic nephrectomy.

## Materials and Methods

### Study Design and Patients

This multicenter research retrospectively collected clinical data from 590 RCC patients who underwent partial or radical nephrectomy at Zhongda Hospital Southeast University, Shanghai Tenth People's Hospital, and Shidong Hospital from January 2014 to December 2019. The inclusion criteria were set as follows: patients with pathologically diagnosed RCC; and patients who received surgical treatment with therapeutic purposes for the first time. The exclusion criteria were set as follows: patients who received other anticancer treatment before nephrectomy, such as transcatheter arterial chemoembolization, radiofrequency ablation, or chemotherapy; patients with other malignant tumors; and patients without complete medical records or lost to follow-up. After screening, this study finally included 443 patients.

A total of 343 patients from Zhongda Hospital Southeast University were included as the training set, and 100 patients from Shanghai Tenth People's Hospital and Shidong Hospital were adopted to the test set. All included patients have signed written informed consent. The methodology of this study followed the criteria outlined in Declaration of Helsinki (as revised in 2013) and was ethically approved by Ethics Committees and Institutional Review Boards of all participating institutions.

### Clinical Data Collection and Follow-Up

Baseline information, laboratory examination, and imaging findings of all patients were reviewed and retrieved from hospital electronic medical records. The collected basic characteristics of patients include age, gender, body mass index [BMI, calculated by weight (kg)/height^2^ (m^2^)], hypertension, diabetes, cardiovascular disease, smoking, surgery type, hemoglobin, ALB, GLB, AGR, AGS, SMI, platelets, neutrophils, lymphocytes, and survival time. Tumor-related clinic pathological features were also collected, including laterality, AJCC stage, TNM stage, and Fuhrman grade. All included patients were followed up to December 2020 by telephone every 3 months. The laboratory test data were measured 2 days before surgery or closest to the time of surgery. Neutrophil to lymphocyte ratio (NLR) is the ratio of neutrophils to lymphocytes, whereas platelet to lymphocyte ratio (PLR) is the ratio of platelets to lymphocytes. AGR is the ratio of serum ALB to GLB. According to previous studies, AGS = 0 means ALB > 41.7 g/L and GLB <28.6 g/L, AGS = 2 means ALB <41.7 g/L and GLB > 28.6 g/L, and AGS = 1 for the remaining patients (8). The diagnosis of sarcopenia was determined based on previous studies (12). CAS was defined as follows: patients with low AGS (AGS = 0) and non-sarcopenia were included in CAS grade 1, patients with high AGS (AGS = 1/2) and sarcopenia were included in CAS grade 3, and the remaining patients were included in CAS grade 2. Overall survival (OS) was calculated from the surgical treatment date to death date or the last follow-up. Cancer-specific survival (CSS) was calculated from the date of therapeutic resection to the date of death due to RCC.

### Statistical Analysis

Continuous data are presented as mean ± standard deviation (SD) and categorical data as number (%). Categorical variables were analyzed using chi-square test or Fisher's exact tests and continuous variables were analyzed using *t*-test. AGR was determined using receiver operating characteristic (ROC) curves and patients were divided into AGR > 1.33 and AGR ≤ 1.33 groups according to AGR levels. Patients with AGS = 0 were included in the low AGS group, and those with AGS = 1 or 2 were included in the high AGS group. We divided patients into sarcopenia and non-sarcopenia groups according to SMI.

Kaplan-Meier curves were employed to assess the effects of AGR, AGS, SMI, and CAS on OS and CSS. ROC curves were utilized to compare the predictive ability of AGR, AGS, SMI, NLR, PLR and CAS on OS and CSS and were numerated using the area under the curve (AUC). Univariate and multivariate Cox regression models were deployed to assess the relationship between CAS and OS and CSS. In multivariate Cox regression analysis, we constructed three models to assess the relationship between CAS and OS and CSS separately and calculated the associated adjusted hazard ratios (aHR) and 95% confidence intervals (CI). In the basic model, we adjusted for age, gender, BMI, hypertension, diabetes, cardiovascular diseases, and smoking. In the core model, we added surgical type and laterality to the seven variables in the base model. In the extended model, we added six variables of AJCC stage, T stage, N stage, M stage, and Fuhrman grade based on the core model. Statistical analysis of this research was performed using SPSS software (version 26.0) and Graphpad Prism (version 8.3.0). A 2-tailed *P* < 0.05 was considered statistically significant.

## Results

**Figure 1:**
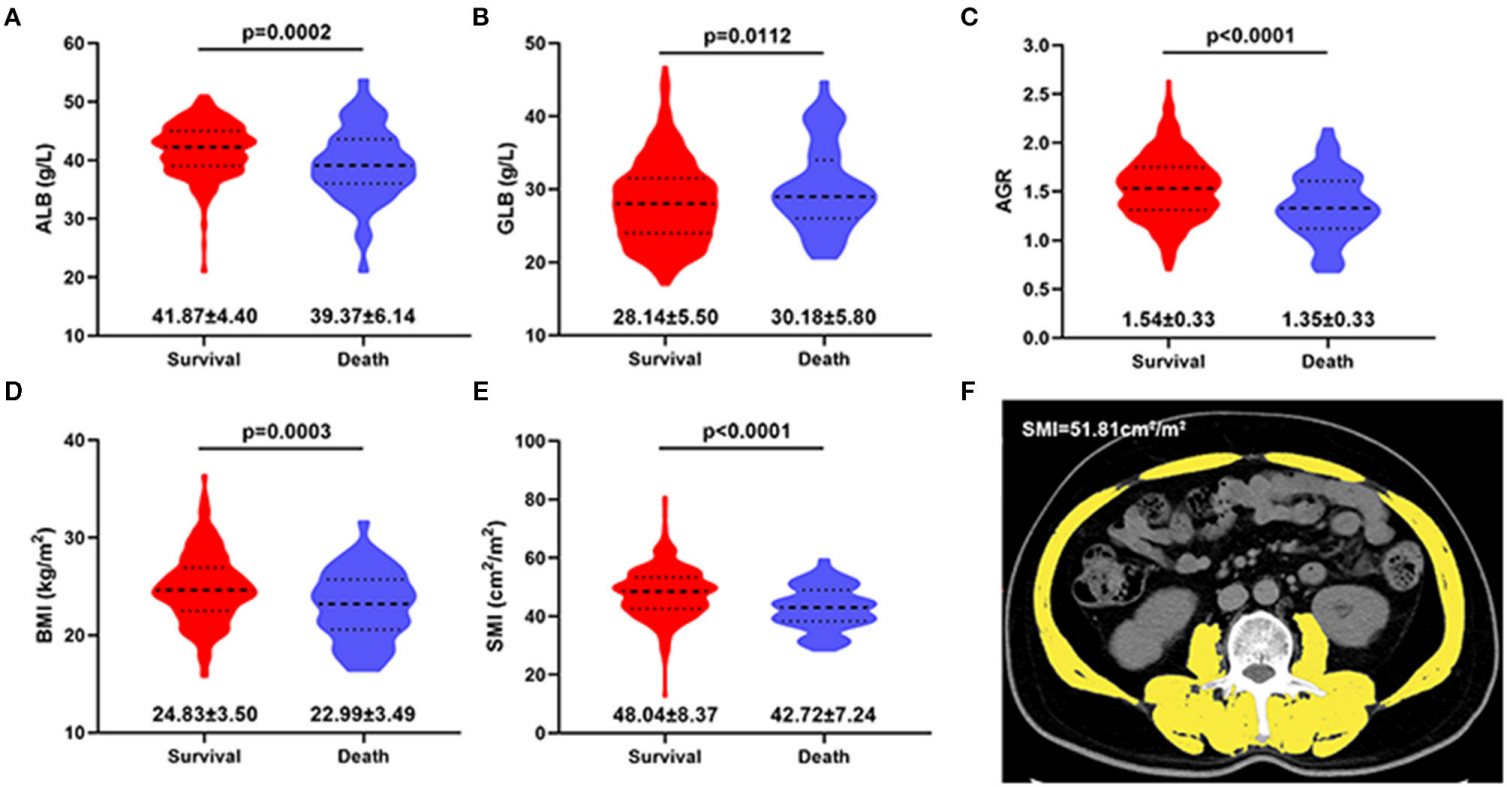
Violin plots showing the preoperative ALB **(A)**, GLB **(B)**, AGR **(C)**, BMI **(D)** and SMI **(E)** level in survival and death group at the end of follow-up. **(F)** Axial CT images of the third lumbar region were used to measure the skeletal muscle index (yellow area). ALB, albumin; GLB, globulin; AGR, albumin to globulin ratio; BMI, Body mass index; SMI, skeletal muscle index. Data are presented as the mean ± standard deviation.

The clinic pathological characteristics of 443 patients included in this study are presented in Table 1. In the entire cohort, the mean age of all patients was 58.02 years, their BMI was 24.60 kg/m^2^, and their survival time was 32.88 months. Preoperative ALB, GLB, AGR, BMI, and SMI levels in surviving patients were higher than those in dead patients (Figure 1). In training and test sets, we found that most patients were male, age <65 years, BMI <25 kg/m^2^, without hypertension or diabetes, or cardiovascular disease. The common tumor types were AJCC I stage, T1 stage, N0 stage, M0 stage, and Fuhrman II grade. In addition, no statistically difference was observed in survival time between patients in training and test sets.

**Table 1:** Baseline characteristics of patients in the training and test sets.

**Characteristic**	**All Patients**	**Training Set**	**Test Set**	***P*-** ** value**
	**No. (%)**	**No. (%)**	**No. (%)**	
Total patients	443	343	100	
Age, y, mean (SD)	58.02 (12.44)	57.47 (12.56)	59.90 (11.89)	0.086
Age categorized, y				0.027
≤ 65	318 (71.8)	255 (74.3)	63 (63.0)	
>65	125 (28.2)	88 (25.7)	37 (37.0)	
Gender				0.442
Male	296 (66.8)	226 (65.9)	70 (70.0)	
Female	147 (33.2)	117 (34.1)	30 (30.0)	
BMI, kg/m^2^, mean (SD)	24.60 (3.55)	24.69 (3.62)	24.30 (3.29)	0.330
BMI categorized, kg/m^2^				0.032
<25	251 (56.7)	185 (53.9)	66 (66.0)	
≥25	192 (43.3)	158 (46.1)	34 (34.0)	
Hypertension				0.444
No	251 (56.7)	191 (55.7)	60 (60.0)	
Yes	192 (43.3)	152 (44.3)	40 (40.0)	
Diabetes				0.993
No	372 (84.0)	288 (84.0)	84 (84.0)	
Yes	71 (16.0)	55 (16.0)	16 (16.0)	
Cardiovascular diseases				0.211
No	392 (88.5)	300 (87.5)	92 (92.0)	
Yes	51 (11.5)	43 (12.5)	8 (8.0)	
Smoking				0.883
No	370 (83.5)	286 (83.4)	84 (84.0)	
Yes	73 (16.5)	57 (16.6)	16 (16.0)	
Surgery type				<0.001
Partial nephrectomy	268 (60.5)	187 (54.5)	81 (81.0)	
Radical nephrectomy	175 (39.5)	156 (45.5)	19 (19.0)	
Laterality				0.580
Left	224 (50.6)	171 (49.9)	53 (53.0)	
Right	219 (49.4)	172 (50.1)	47 (47.0)	
AJCC stage				0.200
I	329 (74.3)	256 (74.6)	73 (73.0)	
II	26 (5.9)	19 (5.5)	7 (7.0)	
III	60 (13.5)	45 (13.1)	15 (15.0)	
IV	28 (6.3)	23 (6.7)	5 (5.0)	
T-stage				1.000
T1	336 (75.8)	260 (75.8)	76 (76.0)	
T2	30 (6.8)	23 (6.7)	7 (7.0)	
T3	66 (14.9)	51 (14.9)	15 (15.0)	
T4	11 (2.5)	9 (2.6)	2 (2.0)	
N-stage				0.590
N0	425 (95.9)	330 (96.2)	95 (95.0)	
N1	18 (4.1)	13 (3.8)	5 (5.0)	
M-stage				0.585
M0	424 (95.7)	327 (95.3)	97 (97.0)	
M1	19 (4.3)	16 (4.7)	3 (3.0)	
Fuhrman grade				0.915
I	74 (16.7)	55 (16.0)	19 (19.0)	
II	276 (62.3)	216 (63.0)	60 (60.0)	
III	83 (18.7)	64 (18.7)	19 (19.0)	
IV	10 (2.3)	8 (2.3)	2 (2.0)	
Hemoglobin (g/L), mean (SD)	133.41 (19.98)	133.14 (20.34)	134.39 (18.77)	0.585
ALB, [g/L, mean (SD)]	41.55 (4.71)	41.12 (4.88)	43.06 (3.74)	<0.001
GLB, [U/L, mean (SD)]	28.40 (5.58)	28.82 (5.72)	26.94 (4.79)	0.003
AGR [mean, (SD)]	1.52 (0.33)	1.48 (0.34)	1.64 (0.27)	<0.001
AGS				0.005
Low (0)	120 (27.1)	82 (23.9)	38 (38.0)	
High (1/2)	323 (72.9)	261 (76.1)	62 (62.0)	
SMI, cm^2^/m^2^, mean (SD)				0.126
Non-sarcopenic	286 (64.6)	215 (62.7)	71 (71.0)	
Sarcopenic	157 (35.4)	128 (37.3)	29 (29.0)	
Survival time (months)	32.88 (19.52)	32.63 (18.90)	33.71 (21.58)	0.628

*Continuous data are presented as the mean ± standard deviation and categorical data as n (%).*

*For categorical variables, P-values were analyzed by chi-square tests. For continuous variables, the t-test for slope was used in generalized linear models. For T-stage, M-stage, and Fuhrman grade, Fisher's exact test was used.*

*SD, standard deviation; BMI, Body mass index; AJCC, American Joint Committee on Cancer; ALB, albumin; GLB, globulin; AGR, albumin to globulin ratio; AGS, albumin-globulin score; SMI, skeletal muscle index*.

**Figure 2:**
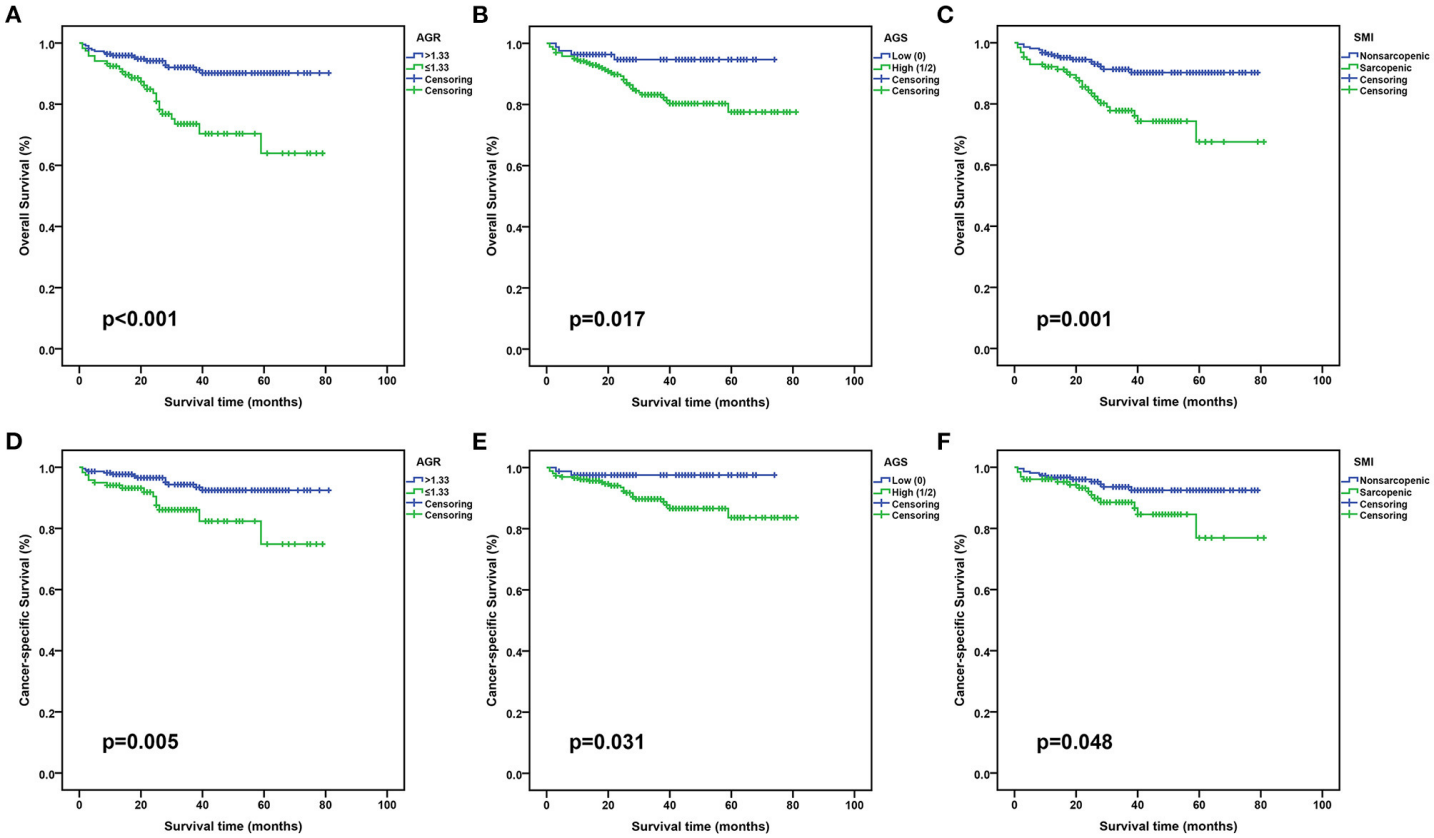
Kaplan-Meier curves for OS and CSS stratified by AGR, AGS and SMI in the training set. **(A,D)**, AGR OS and CSS; **(B,E)**, AGS OS and CSS; **(C,F)**, SMI OS and CSS. OS, overall survival; CSS, cancer-specific survival; AGR, albumin to globulin ratio; AGS, albumin-globulin score; SMI, skeletal muscle index.

As indicated in Table 2, in the training set, 82 (23.9%) patients were classified into low AGS group and 261 (76.1%) patients into high AGS (AGS = 1/2) group according to AGS, while 215 (62.7%) patients had non-sarcopenia and 128 (37.3%) patients had sarcopenia assessed by SMI. Kaplan-Meier survival curves indicated that high AGR, low AGS, and non-sarcopenia predicted higher overall survival (OS) and cancer-specific survival (CSS) in both training and test sets (Figure 2 and Supplementary Figure 1). There was an increased proportion of patients aged >65 years, BMI <25 kg/m^2^ in high AGS group or sarcopenia group. In addition, other variables, such as surgical type, hemoglobin, ALB, GLB, and AGR, were comparable between low and high AGS or sarcopenia and non-sarcopenia groups.

**Table 2:** Comparison between AGS, SMI and clinic pathological characteristics in training set.

**Characteristic**	**AGS**		***P*-value**	**SMI**		***P*-value**
	**Low (0)**	**High (1/2)**		**Non-sarcopenic**	**Sarcopenic**	
	**No. (%)**	**No. (%)**		**No. (%)**	**No. (%)**	
Total patients	82	261		215	128	
Age, y, mean (SD)	53.00 (12.79)	58.87 (12.18)	<0.001	55.73 (11.72)	60.40 (13.40)	0.001
Age categorized, y			0.041			<0.001
≤ 65	68 (82.9)	187 (71.6)		175 (81.4)	80 (62.5)	
>65	14 (17.1)	74 (28.4)		40 (18.6)	48 (37.5)	
Gender			0.289			0.209
Male	58 (70.7)	168 (64.4)		147 (68.4)	79 (61.7)	
Female	24 (29.3)	93 (35.6)		68 (31.6)	49 (38.3)	
BMI, kg/m^2^, mean (SD)	25.07 (3.68)	24.57 (3.60)	0.271	25.38 (3.42)	23.53 (3.66)	<0.001
BMI categorized, kg/m^2^			0.184			0.660
<25	39 (47.6)	146 (55.9)		114 (53.0)	71 (55.5)	
≥25	43 (52.4)	115 (44.1)		101 (47.0)	57 (44.5)	
Hypertension			0.551			0.198
No	48 (58.5)	143 (54.8)		114 (53.0)	77 (60.2)	
Yes	34 (41.5)	118 (45.2)		101 (47.0)	51 (39.8)	
Diabetes			0.769			0.442
No	68 (82.9)	220 (84.3)		178 (82.8)	110 (85.9)	
Yes	14 (17.1)	41 (15.7)		37 (17.2)	18 (14.1)	
Cardiovascular diseases			0.383			0.319
No	74 (90.2)	226 (86.6)		191 (88.8)	109 (85.2)	
Yes	8 (9.8)	35 (13.4)		24 (11.2)	19 (14.8)	
Smoking			0.831			0.604
No	69 (84.1)	217 (83.1)		181 (84.2)	105 (82.0)	
Yes	13 (15.9)	44 (16.9)		34 (15.8)	23 (18.0)	
Surgery type			0.018			0.002
Partial nephrectomy	54 (65.9)	133 (51.0)		131 (60.9)	56 (43.8)	
Radical nephrectomy	28 (34.1)	128 (49.0)		84 (39.1)	72 (56.2)	
Laterality			0.217			0.282
Left	46 (56.1)	126 (48.3)		103 (47.9)	69 (53.9)	
Right	36 (43.9)	135 (51.7)		112 (52.1)	59 (46.1)	
AJCC stage			0.484			0.749
I	67 (81.7)	189 (72.4)		163 (75.8)	93 (72.7)	
II	3 (3.7)	16 (6.1)		13 (6.0)	6 (4.7)	
III	8 (9.8)	37 (14.2)		26 (12.1)	19 (14.8)	
IV	4 (4.9)	19 (7.3)		13 (6.0)	10 (7.8)	
T-stage			0.334			0.530
T1	67 (81.7)	193 (73.9)		166 (77.2)	94 (73.4)	
T2	4 (4.9)	19 (7.3)		16 (7.4)	7 (5.5)	
T3	8 (9.8)	43 (16.5)		28 (13.0)	23 (18.0)	
T4	3 (3.7)	6 (2.3)		5 (2.3)	4 (3.1)	
N-stage			0.316			0.774
N0	81 (98.8)	249 (95.4)		206 (95.8)	124 (96.9)	
N1	1 (1.2)	12 (4.6)		9 (4.2)	4 (3.1)	
M-stage			0.376			0.604
M0	80 (97.6)	247 (94.6)		206 (95.8)	121 (94.5)	
M1	2 (2.4)	14 (5.4)		9 (4.2)	7 (5.5)	
Fuhrman grade			0.437			0.708
I	15 (18.3)	40 (15.3)		38 (17.7)	17 (13.3)	
II	53 (64.6)	163 (62.5)		134 (62.3)	82 (64.1)	
III	14 (17.1)	50 (19.2)		38 (17.7)	26 (20.3)	
IV	0 (0.0)	8 (3.1)		5 (2.3)	3 (2.3)	
Hemoglobin (g/L), mean (SD)	140.46 (16.10)	130.84 (21.00)	<0.001	135.40 (18.60)	129.33 (22.52)	0.007
ALB, [g/L, mean (SD)]	44.79 (2.22)	39.93 (5.08)	<0.001	41.57 (4.97)	40.29 (4.99)	0.022
GLB, [U/L, mean (SD)]	24.35 (2.85)	30.22 (5.68)	<0.001	28.75 (5.72)	28.93 (5.73)	0.782
AGR [mean, (SD)]	1.87 (0.25)	1.36 (0.28)	<0.001	1.50 (0.34)	1.45 (0.35)	0.186
Survival time (months)	35.48 (19.44)	31.74 (18.68)	0.119	32.84 (19.50)	32.28 (17.92)	0.791

*Continuous data are presented as the mean ± standard deviation and categorical data as n (%).*

*For categorical variables, P-values were analyzed by chi-square tests. For continuous variables, the t-test for slope was used in generalized linear models. For AJCC stage, T-stage, N-stage, M-stage, and Fuhrman grade, Fisher's exact test was used.*

*SD, standard deviation; BMI, Body mass index; AJCC, American Joint Committee on Cancer; ALB, albumin; GLB, globulin; AGR, albumin to globulin ratio; AGS, albumin-globulin score; SMI, skeletal muscle index*.

**Figure 3:**
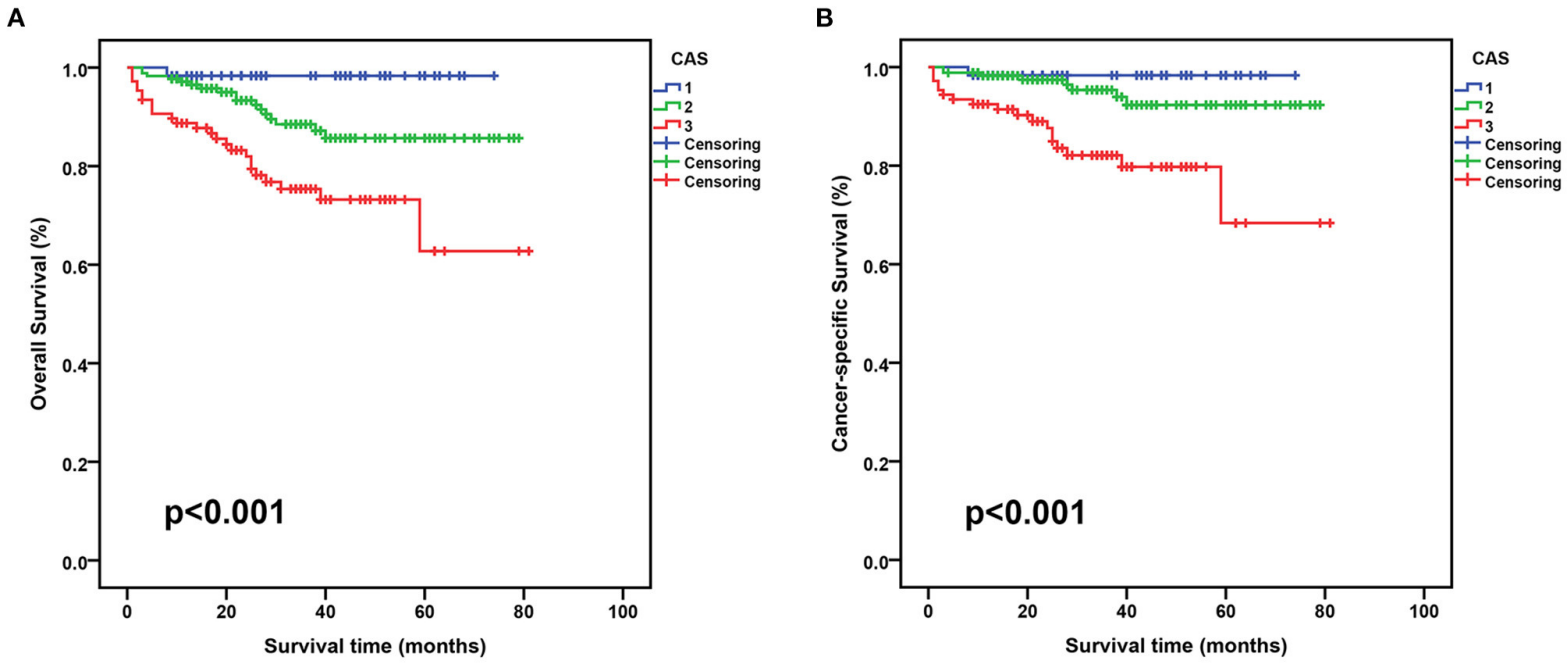
Kaplan-Meier curves for OS and CSS stratified by CAS grade in the training set. **(A)**, CAS OS; **(B)**, CAS CSS. OS, overall survival; CSS, cancer-specific survival; CAS, combination of albumin-globulin score and sarcopenia.

When stratified by CAS grade, 60 (17.5%) patients were CAS grade 1, 176 (51.3%) patients were CAS grade 2, and 107 (31.2%) patients were CAS grade 3. Table 3 displays the relationship between CAS and patient clinicopathology. We found that CAS grade 3 group had a higher percentage of age >65 years, female, BMI <25 kg/m^2^, AJCC III/IV stage, T3–4 stage, N1 stage, M1 stage, and Fuhrman III/IV grade than those in the other two groups. In training and test sets, survival time progressively decreased with increasing CAS grade, and patients with CAS grade 3 were associated with the lowest OS and CSS (Figure 3 and Supplementary Figure 2). In addition, statistical differences existed among the three groups in age, BMI, surgical type, hemoglobin, ALB, GLB, and AGR variables.

**Table 3:** Comparison between CAS and clinic pathological characteristics in training set.

**Characteristic**	**CAS**			***P*-value**
	**Grade 1**	**Grade 2**	**Grade 3**	
	**No. (%)**	**No. (%)**	**No. (%)**	
Total patients	60 (17.5)	176 (51.3)	107 (31.2)	
Age, y, mean (SD)	51.23 (12.56)	57.52 (11.05)	60.89 (13.63)	<0.001
Age categorized, y				<0.001
≤ 65	50 (83.3)	141 (80.1)	64 (59.8)	
>65	10 (16.7)	35 (19.9)	43 (40.2)	
Gender				0.180
Male	42 (70.0)	121 (68.8)	63 (58.9)	
Female	18 (30.0)	55 (31.2)	44 (41.1)	
BMI, kg/m^2^, mean (SD)	25.72 (3.51)	25.05 (3.45)	23.52 (3.68)	<0.001
BMI categorized, kg/m^2^				0.310
<25	27 (45.0)	98 (55.7)	60 (56.1)	
≥25	33 (55.0)	78 (44.3)	47 (43.9)	
Hypertension				0.946
No	33 (55.0)	97 (55.1)	61 (57.0)	
Yes	27 (45.0)	79 (44.9)	46 (43.0)	
Diabetes				0.430
No	48 (80.0)	152 (86.4)	88 (82.2)	
Yes	12 (20.0)	24 (13.6)	19 (17.8)	
Cardiovascular diseases				0.261
No	53 (88.3)	158(89.8)	89 (83.2)	
Yes	7 (11.7)	18 (10.2)	18 (16.8)	
Smoking				0.742
No	52 (86.7)	145 (82.4)	89 (83.2)	
Yes	8 (13.3)	31 (17.6)	18 (16.8)	
Surgery type				<0.001
Partial nephrectomy	45 (75.0)	96 (54.5)	46 (43.0)	
Radical nephrectomy	15 (25.0)	80 (45.5)	61 (57.0)	
Laterality				0.834
Left	32 (53.3)	86 (48.9)	54 (50.5)	
Right	28 (46.7)	90 (51.1)	53 (49.5)	
AJCC stage				0.237
I	49 (81.7)	134 (76.1)	73 (68.2)	
II	2 (3.3)	11 (6.2)	6 (5.6)	
III	5 (8.3)	24 (13.6)	16 (15.0)	
IV	4 (6.7)	7 (4.0)	12 (11.2)	
T-stage				0.155
T1	49 (81.7)	137 (77.8)	74 (69.2)	
T2	3 (5.0)	13 (7.4)	7 (6.5)	
T3	5 (8.3)	24 (13.6)	22 (20.6)	
T4	3 (5.0)	2 (1.1)	4 (3.7)	
N-stage				0.644
N0	59 (98.3)	169 (96.0)	102 (95.3)	
N1	1 (1.7)	7 (4.0)	5 (4.7)	
M-stage				0.102
M0	58 (96.7)	171 (97.2)	98 (91.6)	
M1	2 (3.3)	5 (2.8)	9 (8.4)	
Fuhrman grade				**0.160**
I	10 (16.7)	34 (19.3)	11 (10.3)	
II	39 (65.0)	111 (63.1)	66 (61.7)	
III	11 (18.3)	28 (15.9)	25 (23.4)	
IV	0 (0.0)	3 (1.7)	5 (4.7)	
Hemoglobin (g/L), mean (SD)	140.90 (17.16)	135.28 (16.79)	125.26 (24.51)	<0.001
ALB, [g/L, mean (SD)]	44.93 (2.29)	40.88 (4.89)	39.27 (5.17)	<0.001
GLB, [U/L, mean (SD)]	24.39 (2.77)	29.43 (5.61)	30.30 (5.95)	<0.001
AGR [mean, (SD)]	1.87 (0.25)	1.43 (0.29)	1.35 (0.31)	<0.001
Survival time (months)	35.17 (18.80)	33.10 (19.58)	30.44 (17.73)	0.106

*Continuous data are presented as the mean ± standard deviation and categorical data as n (%).*

*For categorical variables, P-values were analyzed by chi-square tests. For continuous variables, the t-test for slope was used in generalized linear models. For AJCC stage, T-stage, N-stage, M-stage, and Fuhrman grade, Fisher's exact test was used.*

*SD, standard deviation; BMI, Body mass index; AJCC, American Joint Committee on Cancer; ALB, albumin; GLB, globulin; AGR, albumin to globulin ratio; CAS, combination of albumin-globulin score and skeletal muscle index*.

In addition, we constructed three multivariate Cox regression models to assess the correlation of CAS with OS and CSS (Table 4). The results revealed that CAS was consistently an independent risk factor for OS (extended model: CAS grade 1 vs. CAS grade 3: aHR = 0.08; 95% CI: 0.01–0.58, *p* = 0.012; CAS grade 2 vs. CAS grade 3: aHR = 0.47; 95% CI: 0.25–0.88, *p* = 0.018) and CSS (extended model: CAS grade 1 vs. CAS grade 3: aHR = 0.12; 95% CI: 0.02-0.94, *p* = 0.043; CAS grade 2 vs. CAS grade 3: aHR = 0.31; 95% CI: 0.13–0.71, *p* = 0.006), whether in the basic, core, or extended models and CAS grade 3 was associated with the worst prognosis.

**Table 4:** Hazard ratios of overall survival (OS) and cancer-specific survival (CSS) was calculated according to CAS in training set^a^.

**Characteristic**	**Basic Model**		**Core Model**		**Extended Model**	
	**aHR (95% CI)**	***P*-value**	**aHR (95% CI)**	***P-*value**	**aHR (95% CI)**	***P-*value**
**Overall Survival**						
CAS		<0.001		<0.001		<0.001
Grade 1	0.07 (0.01–0.48)	0.007	0.09 (0.01–0.67)	0.018	0.08 (0.01–0.58)	0.012
Grade 2	0.40 (0.21–0.73)	0.003	0.43 (0.23–0.80)	0.008	0.47 (0.25–0.88)	0.018
Grade 3	Reference		Reference		Reference	
**Cancer-specific Survival**						
CAS		<0.001		0.001		0.003
Grade 1	0.09 (0.01–0.67)	0.019	0.14 (0.02–0.93)	0.042	0.12 (0.02–0.94)	0.043
Grade 2	0.26 (0.11–0.59)	0.001	0.28 (0.12–0.66)	0.003	0.31 (0.13–0.71)	0.006
Grade 3	Reference		Reference		Reference	

a *Adjusted covariates: Basic model: age, gender, BMI, hypertension, diabetes, cardiovascular diseases and smoking; Core model: basic model plus surgery type and laterality; Extended model: core model plus AJCC stage, T stage, N stage, M stage and Fuhrman grade.*

*BMI, Body mass index; AJCC, American Joint Committee on Cancer; aHR, adjusted hazard ratio; CI, confidence interval; CAS, combination of albumin-globulin score and skeletal muscle index*.

**Figure 4:**
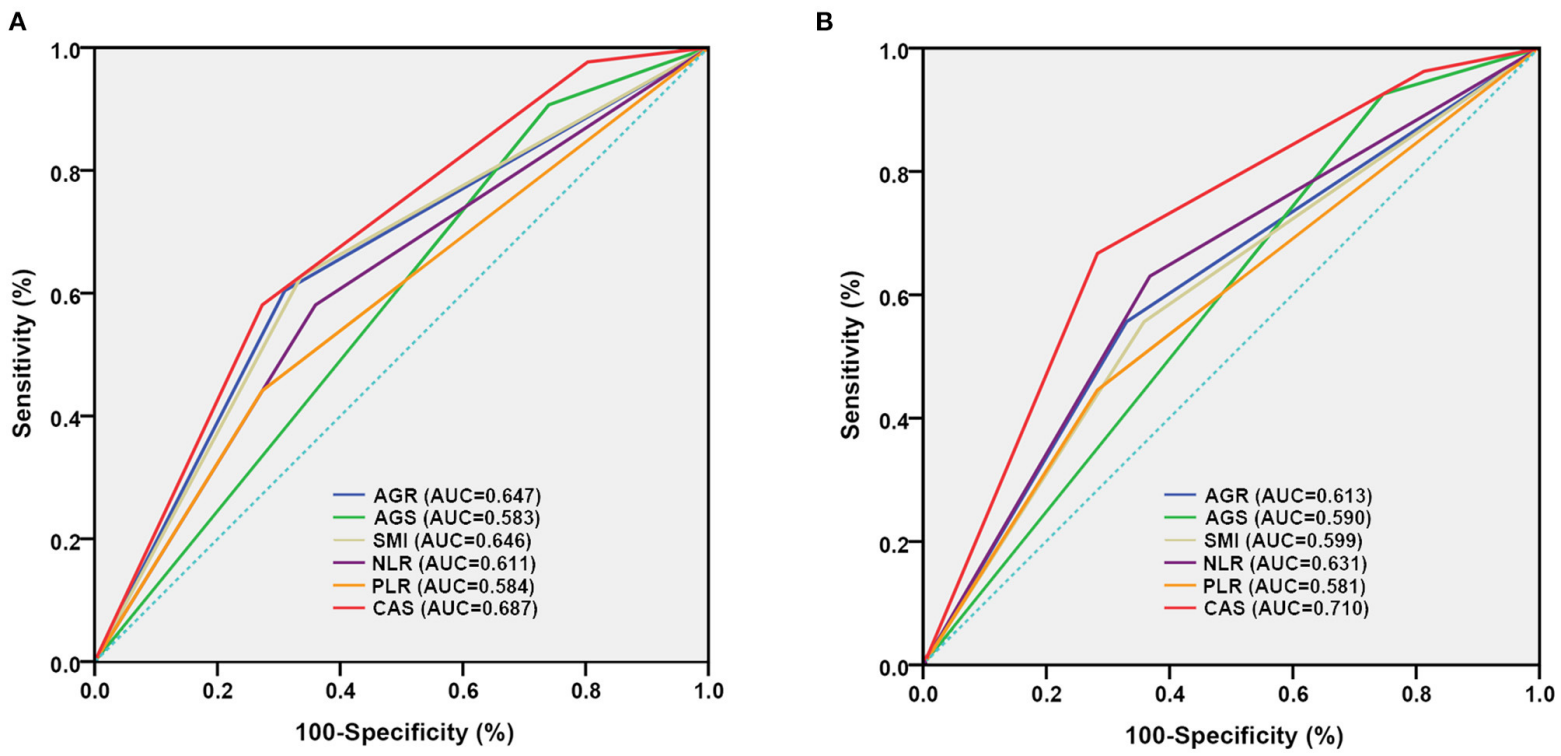
Comparison of area under ROC curves for AGR, AGS, SMI and CAS grade in predicting OS and CSS in the training set. **(A)**, OS ROC curves; **(B)**, CSS ROC curves. OS, overall survival; CSS, cancer-specific survival; ROC, receiver operator characteristic; AUC, area under the curve; AGR, albumin to globulin ratio; AGS, albumin-globulin score; SMI, skeletal muscle index; CAS, combination of albumin-globulin score and sarcopenia.

Receiver operating characteristic (ROC) curves were utilized to evaluate the prognostic ability of AGR, AGS, SMI, NLR, PLR, and CAS in RCC patients undergoing laparoscopic nephrectomy (Table 5). We discovered that CAS had higher predictive power for OS (training set: AUC = 0.687, 95% CI: 0.607–0.766, *p* < 0.001; test set: AUC = 0.724, 95% CI: 0.557–0.891, *p* = 0.012) and CSS (training set: AUC = 0.710, 95% CI: 0.613–0.808, *p* < 0.001; test set: AUC = 0.805, 95% CI: 0.648–0.962, *p* = 0.004) than the other five indicators in training and test sets (Figure 4 and Supplementary Figure 3).

**Table 5:** Accuracy of AGR, AGS, SMI and CAS in predicting overall survival (OS) and cancer-specific survival (CSS) by assessing the area under the curve (AUC) in the training and test sets.

**Characteristics**		**Training Set**			**Test Set**		
		**AUC**	**95% CI**	***P*-value**	**AUC**	**95% CI**	***P*-value**
Overall survival	AGR	0.647	0.557–0.737	0.002	0.504	0.330–0.678	0.966
	AGS	0.583	0.500–0.667	0.077	0.527	0.354–0.699	0.766
	SMI	0.646	0.556–0.735	0.002	0.714	0.550–0.878	0.017
	NLR	0.611	0.520–0.702	0.019	0.477	0.305–0.649	0.799
	PLR	0.584	0.490–0.679	0.074	0.566	0.406–0.727	0.458
	CAS	0.687	0.607–0.766	<0.001	0.724	0.557–0.891	0.012
Cancer-specific survival	AGR	0.613	0.500–0.726	0.051	0.481	0.266–0.696	0.859
	AGS	0.590	0.490–0.689	0.122	0.571	0.372–0.769	0.509
	SMI	0.599	0.486–0.712	0.088	0.750	0.568–0.932	0.019
	NLR	0.631	0.522–0.741	0.024	0.478	0.272–0.684	0.703
	PLR	0.581	0.465–0.698	0.160	0.541	0.342–0.740	0.839
	CAS	0.710	0.613–0.808	<0.001	0.805	0.648–0.962	0.004

*OS, overall survival; CSS, cancer-specific survival; CI, confidence interval; AGR, albumin to globulin ratio; AGS, albumin-globulin score; SMI, skeletal muscle index; CAS, combination of albumin-globulin score and skeletal muscle index*.

## Discussion

In the current study, given the prognostic value of ALB, GLB, and sarcopenia in RCC patients, we combined them to construct a new index (CAS), providing a more comprehensive response to systemic nutritional and inflammatory status. CAS has been demonstrated to have a predictive role in patients with intrahepatic cholangiocarcinoma (ICC). By retrospectively analyzing clinical data from 613 ICC patients, Li et al. (14) found that CAS was strongly associated with long-term postoperative outcomes for surgically treated ICC patients. We conducted a multicenter study to investigate the impact of CAS on the prognosis of RCC patients undergoing nephrectomy. This study revealed that a high CAS grade was associated with a poor prognosis. CAS was an independent prognostic risk factor for OS and CSS in RCC patients, and CAS had higher accuracy in predicting OS and CSS than AGR, AGS, and sarcopenia.

ALB and GLB are two important components of human serum proteins. Serum albumin is frequently used to determine the nutritional status of patients (15). Hypoalbuminemia in cancer patients is not only an indicator of malnutrition but is also associated with a systemic inflammatory response, which may be caused by cytokine-induced immunosuppression (16). Recent research has demonstrated that serum ALB levels can predict the prognosis of cancer patients. In addition, combination factors consisting of ALB and other indicators (such as C-reactive protein) can predict the prognosis of RCC patients (17, 18).

GLB is the major component of non-albumin proteins in serum, and the serum GLB component contains various proteins that are critical in immune and inflammatory responses, including immunoglobulins, complement, and some acute-phase response proteins (C-reactive protein, cytokines, etc.) (19). Increased GLB levels can be considered a marker of chronic inflammatory response, reflecting the accumulation of various pro-inflammatory cytokines (20).

Since ALB levels are associated with many factors, such as stress, liver insufficiency, and changes in body fluid volume, their clinical utility for predicting cancer patient prognosis is limited (21). In contrast, AGR is unaffected by the aforementioned factors. AGR is a new tumor predictor for upper tract urothelial carcinoma, esophageal squamous cell carcinoma, non-small cell lung cancer and other tumors (22–25). Meanwhile, the albumin-globulin score (AGS) has been proposed as another prognostic model to predict the prognosis of certain tumors, such as non-small cell lung cancer and esophageal squamous carcinoma (8, 26).

Sarcopenia is not a simple loss of weight or slimming tissue, but a progressive and widespread loss of skeletal muscle mass, strength, and body skeletal muscle. As the decline of skeletal muscle mass may be reversible, sarcopenia has important implications for guiding clinical practice (27). Some studies have demonstrated that establishing a regular exercise and nutritional support program before operation can lead to increased daily calorie and protein intake, as well as a significant increase in grip strength (28, 29). In the study, we found that 44.5% of the sarcopenia patients had BMI ≥ 25 kg/m^2^. The coexistence of obesity and sarcopenia is increasing, and these people are also at risk of their complications (30). Additionally, sarcopenia is more prevalent in elderly patients, contributing to sarcopenia patients' increased risk of death. For lean patients with low BMI, early intervention and increased dietary supplements with protein, vitamin D and antioxidants can slow sarcopenia progression (31, 32).

To our knowledge, this is the first multicenter clinical study to explore the prognostic value of CAS in RCC patients undergoing nephrectomy. For calculating CAS grade, ALB, GLB, and SMI for calculating sarcopenia are more readily available clinically and less costly. In addition, CAS grade combines three indicators, ALB, GLB, and sarcopenia, to accurately reflect patients' nutritional and inflammatory status and expand the predictive ability of individual indicators of ALB, GLB, or sarcopenia for RCC patients.

This study also has several limitations. First, we excluded other treatment modalities, which will have an impact on prognosis. Second, we did not assess the patient's quality of life, energy level, and postoperative nutritional status. Finally, although this is a multicenter study, it remained a retrospective study which requires a larger sample size than a prospective study.

## Conclusion

We successfully constructed an index (CAS) that can more accurately predict the prognosis of RCC patients undergoing laparoscopic nephrectomy.

## Data Availability Statement

Publicly available datasets were analyzed in this study. This data can be found here. The datasets used and analyzed during the current study are available from the corresponding author on reasonable request.

## Ethics Statement

The studies involving human participants were reviewed and approved by The methodology of this study was ethically approved by the Ethics Committees and Institutional Review Boards of all participating institutions (SHSY-IEC-BG/02.04/04.0-81602469 and ZDKYSB077). The patients/participants provided their written informed consent to participate in this study. Written informed consent was obtained from the individual(s) for the publication of any potentially identifiable images or data included in this article.

## Author Contributions

WM, JW, HZ, and MC conception and design. BX and MC administrative support. SS, ZX, JY, CW, SC, and BX collection and assembly of data. WM and KW data analysis and interpretation. WM, NZ, KW, and QH manuscript writing. All authors are final approval of manuscript.

## Funding

This study was supported by the National Natural Science Foundation of China (81672551), the Scientific Research Foundation of Graduate School of Southeast University (YBPY2173), Postgraduate Research & Practice Innovation Program of Jiangsu Province (KYCX21_0156), Jiangsu Provincial Key Research and Development Program (BE2019751), Innovative Team of Jiangsu Provincial (2017XKJQW07), and The National Key Research and Development Program of China (SQ2017YFSF090096), and the Fundamental Research Funds for the Central Universities (2242021S40011).

## Conflict of Interest

The authors declare that the research was conducted in the absence of any commercial or financial relationships that could be construed as a potential conflict of interest.

## Publisher's Note

All claims expressed in this article are solely those of the authors and do not necessarily represent those of their affiliated organizations, or those of the publisher, the editors and the reviewers. Any product that may be evaluated in this article, or claim that may be made by its manufacturer, is not guaranteed or endorsed by the publisher.

## Supplementary Material

Supplementary Figure 1 Kaplan-Meier curves for OS and CSS stratified by AGR, AGS and SMI in the test set. **A** and **D**, AGR OS and CSS; **B** and **E**, AGS OS and CSS; **C** and **F**, SMI OS and CSS. OS, overall survival; CSS, cancer-specific survival; AGR, albumin to globulin ratio; AGS, albumin-globulin score; SMI, skeletal muscle index.

Supplementary Figure 2 Kaplan-Meier curves for OS and CSS stratified by CAS grade in the test set. **A**, CAS OS; **B**, CAS CSS. OS, overall survival; CSS, cancer-specific survival; CAS, combination of albumin-globulin score and sarcopenia.

Supplementary Figure 3 Comparison of area under ROC curves for AGR, AGS, SMI and CAS grade in predicting OS and CSS in the test set. **A**, OS ROC curves; **B**, CSS ROC curves. OS, overall survival; CSS, cancer-specific survival; ROC, receiver operator characteristic; AUC, area under the curve; AGR, albumin to globulin ratio; AGS, albumin-globulin score; SMI, skeletal muscle index; CAS, combination of albumin-globulin score and sarcopenia.
