# Supplementary figures and images for: Combination of Albumin-Globulin Score and Sarcopenia to Predict Prognosis in Patients With Renal Cell Carcinoma Undergoing Laparoscopic Nephrectomy
Source: Front Nutr. 2021 Sep 23;8:731466. doi: 10.3389/fnut.2021.731466 (PMC8495413; doi:10.3389/fnut.2021.731466)

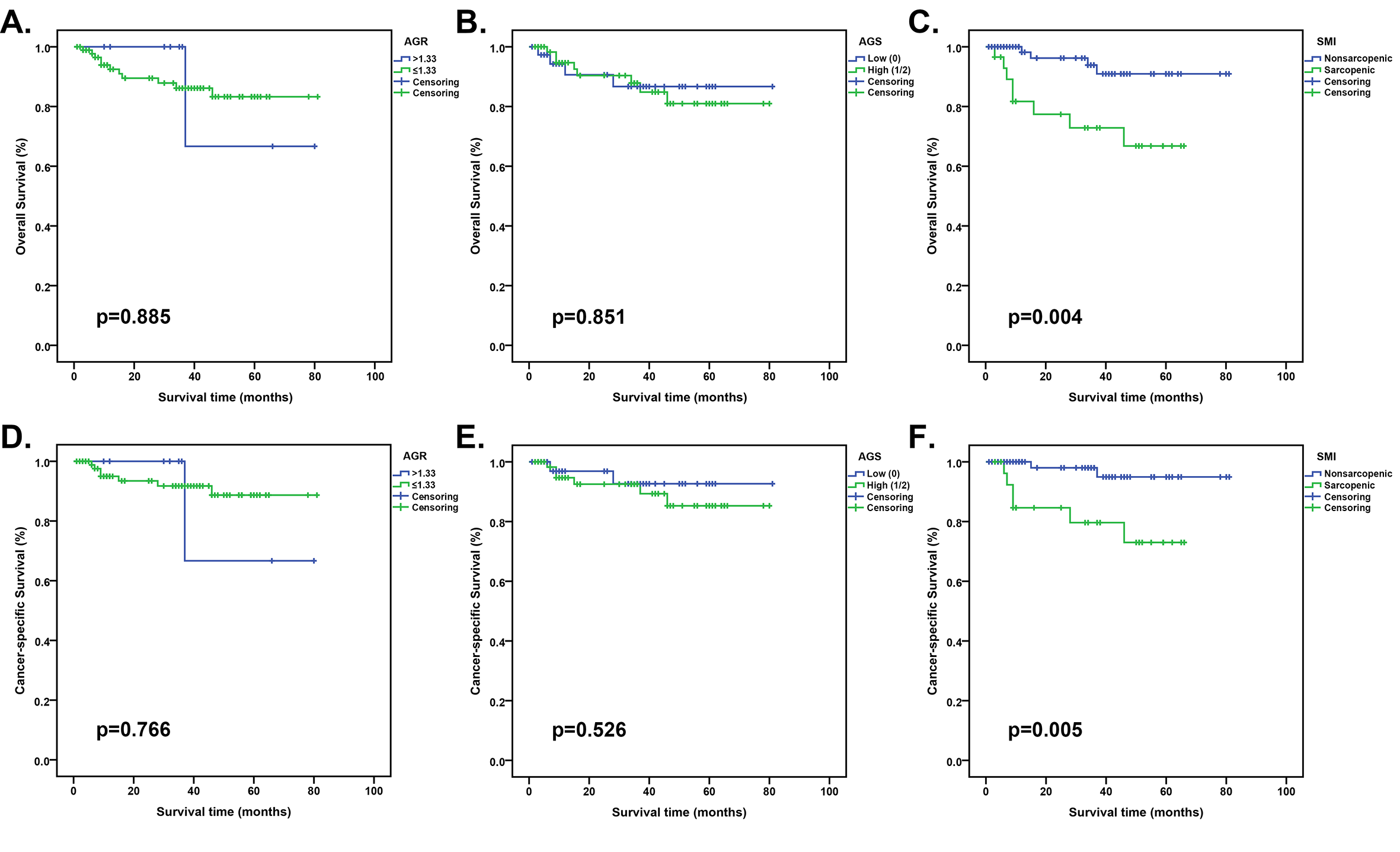

Supplement: Supplementary Figure 1 — Kaplan-Meier curves for OS and CSS stratified by AGR, AGS and SMI in the test set. A and D, AGR OS and CSS; B and E, AGS OS and CSS; C and F, SMI OS and CSS. OS, overall survival; CSS, cancer-specific survival; AGR, albumin to globulin ratio; AGS, albumin-globulin score; SMI, skeletal muscle index. [file Image_1.TIF]

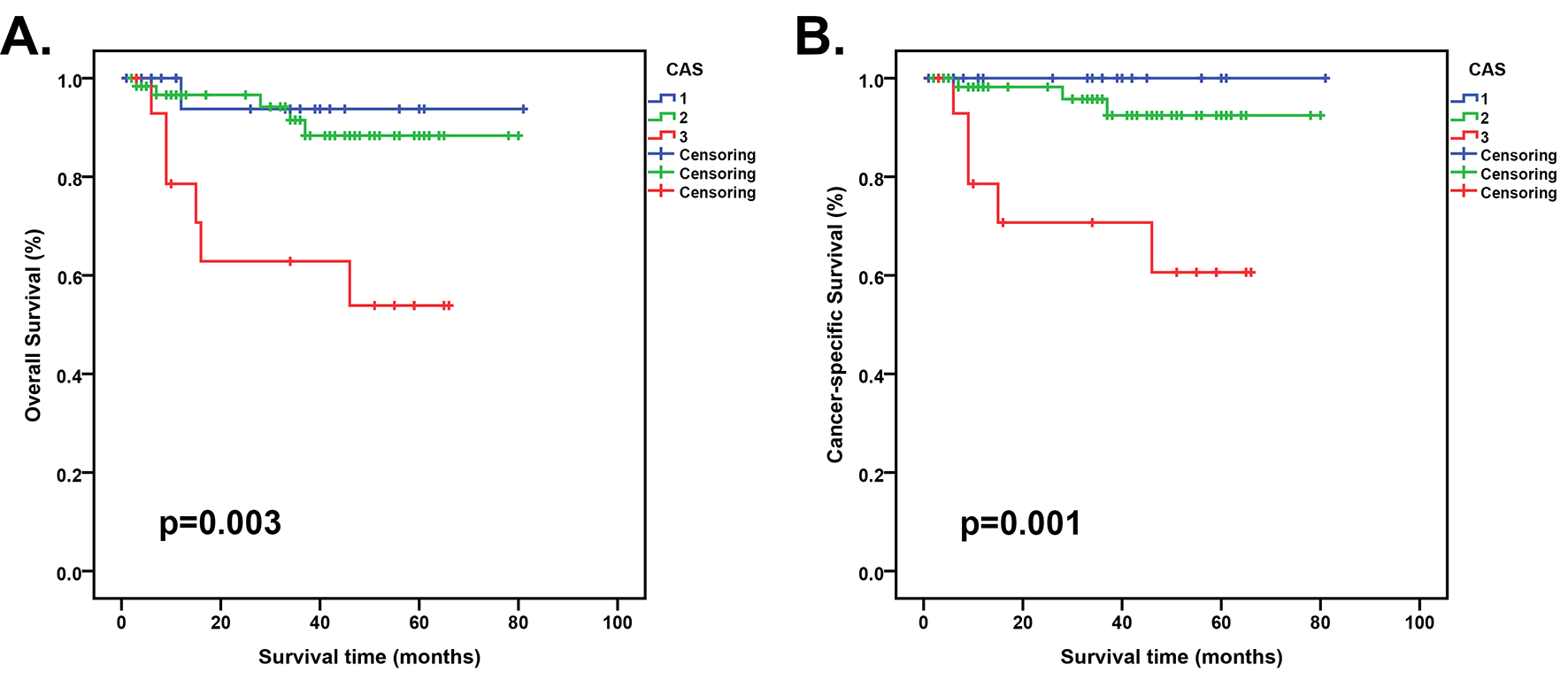

Supplement: Supplementary Figure 2 — Kaplan-Meier curves for OS and CSS stratified by CAS grade in the test set. A, CAS OS; B, CAS CSS. OS, overall survival; CSS, cancer-specific survival; CAS, combination of albumin-globulin score and sarcopenia. [file Image_2.TIF]

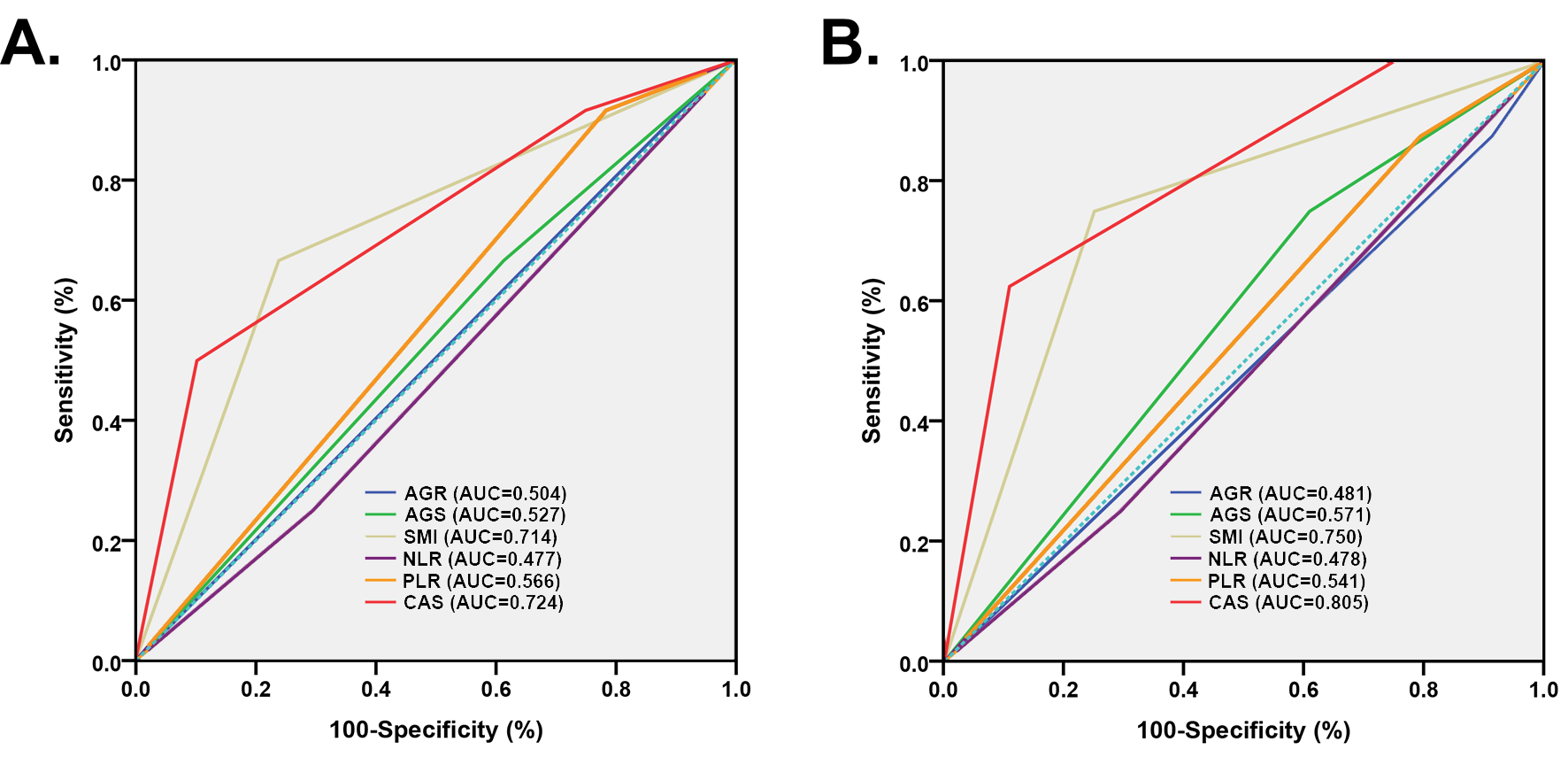

Supplement: Supplementary Figure 3 — Comparison of area under ROC curves for AGR, AGS, SMI and CAS grade in predicting OS and CSS in the test set. A, OS ROC curves; B, CSS ROC curves. OS, overall survival; CSS, cancer-specific survival; ROC, receiver operator characteristic; AUC, area under the curve; AGR, albumin to globulin ratio; AGS, albumin-globulin score; SMI, skeletal muscle index; CAS, combination of albumin-globulin score and sarcopenia. [file Image_3.TIF]
